# N-carbamylglutamate supplementation regulates hindgut microbiota composition and short-chain fatty acid contents in Charollais and Small Tail Han crossbred sheep

**DOI:** 10.3389/fvets.2023.1230190

**Published:** 2023-09-20

**Authors:** Wei Ma, Meiling Yuan, Shuai Chang, Chunqiang Wang

**Affiliations:** College of Animal Science and Veterinary Medicine, Jinzhou Medical University, Jinzhou, China

**Keywords:** N-carbamylglutamate, mutton sheep, hindgut microbiota, hindgut fermentation, short-chain fatty acid

## Abstract

**Introduction:**

The objective of this study was to investigate the effects of N-carbamylglutamate (NCG) supplementation on the growth performance, hindgut microbiota composition, and short-chain fatty acid (SCFA) contents in Charollais and Small Tail Han crossbred sheep.

**Methods:**

A total of 16 female crossbred mutton sheep (Charollais × Small Tail Han), aged 4 months, with an initial body weight of 30.03 ± 0.08 kg, were utilized in a 60 days experiment. The sheep were divided into two groups based on their initial body weight. Each group consisted of 8 replicates, with each individual sheep considered as a replicate. The dietary treatments comprised a basal diet supplemented with either 0.00% or 0.12% NCG.

**Results and discussion:**

Our findings indicate that NCG supplementation did not have a significant effect on the growth performance of mutton sheep. However, it did lead to changes in hindgut SCFA contents. Specifically, NCG supplementation increased the content of propanoic acid while decreasing acetic acid and hexanoic acid in the hindgut. Through microbiota analysis using the *16S rRNA* technique, we identified *Lachnospiraceae_NK3A20_group* and *Parasutterella* as biomarkers for the hindgut microbiota in mutton sheep fed a diet containing NCG. Further analysis of the microbiota composition revealed that NCG supplementation significantly increased the abundance of *Lachnospiraceae_NK3A20_group* and *Parasutterella*, while decreasing *unclassified_f_Lachnospiraceae* and *Lachnoclostridium*. Correlation analysis between hindgut SCFA contents and microbiota composition revealed that the abundance of *Lachnoclostridium* was positively correlated with the contents of acetic acid and hexanoic acid, but negatively correlated with propanoic acid. Additionally, the abundance of *Lachnospiraceae_NK3A20_group* and *Parasutterella* was positively correlated with the content of propanoic acid, while being negatively correlated with acetic acid and hexanoic acid. Based on these findings, we conclude that dietary supplementation of 0.12% NCG can modulate hindgut SCFA contents in mutton sheep by regulating the composition of the hindgut microbiota.

## Introduction

In recent years, there has been a growing interest among animal nutritionists in studying the intestinal microbiota due to its close relationship with the growth and production performance of animals (1). The intestinal microbiota thrives in the nutrient-rich environment provided by the intestine and plays a pivotal role in modulating various host functions, including energy homeostasis, nutrient processing, and immune system development. Bacteria within the intestine produce proteins and metabolites that are influenced by the diet, and among them, short-chain fatty acids (SCFA) hold particular importance in maintaining intestinal homeostasis. SCFA are generated through the bacterial fermentation of dietary fibers and serve as essential fuels for intestinal epithelial cells. They regulate the functions, proliferation, and differentiation of intestinal epithelial cells, thus significantly influencing intestinal function and host metabolism (2).

Given the significant impact of diet on the composition of the intestinal microbiota, animal nutritionists often explore strategies to modulate the intestinal microbiota by providing animals with different types of exogenous additives (3). The objective is to identify suitable additives that can effectively regulate the production of intestinal SCFA by modulating the intestinal microbiota and subsequently improving the animals’ growth performance. N-carbamylglutamate (NCG) is one such additive that has demonstrated efficacy in enhancing endogenous arginine synthesis (4). Acting as a functional analog of N-acetylglutamate, NCG helps restore or improve urea cycle function (5). By entering the mitochondria and activating the key enzyme carbamoyl phosphate synthase-1 in the urea cycle, NCG accelerates citrulline synthesis and promotes endogenous arginine production (6). In monogastric animals, NCG supplementation has been reported to improve growth and reproductive performance (7–9).

While limited research has investigated the effects of NCG supplementation on hindgut SCFA contents and the intestinal microbiota in ruminants, some studies have provided promising insights. For instance, Buchon et al. (10) found that rabbits fed a diet supplemented with NCG exhibited improved development of the jejunum and ileum. Zhang et al. (11) supplemented NCG in the diet of Tan sheep and observed a significant increase in carcass weight and net meat weight. Additionally, Zhao and Zhong (12) reported that daily supplementation of NCG in cows’ feed improved their antioxidant capacity. These findings suggest that NCG may function as a suitable exogenous additive for enhancing the performance of ruminants. In comparison to other crossbred sheep, the Charollais and Small Tail Han crossbred sheep have demonstrated excellent reproductive performance and disease resistance (13). However, improvements are needed in their growth performance. Given the significance of hindgut SCFA contents and intestinal microbiota for animal health, further investigation is warranted to explore the effects of NCG supplementation on the growth performance, hindgut microbiota composition, and SCFA contents of Charollais × Small Tail Han crossbred sheep. We hypothesize that NCG supplementation will increase hindgut SCFA contents in mutton sheep by modulating the hindgut microbiota composition, potentially resulting in enhanced growth performance.

## Materials and methods

### Animals and experimental design

A total of 16 healthy female crossbred mutton sheep (Charolais × Small Tail Han), aged 4 months, with similar initial body weight (30.03 ± 0.08 kg), were utilized in a 60 days experiment. The sheep were divided into two groups based on their initial body weight, with 8 replicates per group, and each sheep served as a replicate. The dietary treatments consisted of a basal diet supplemented with either 0.00% or 0.12% NCG. The dosage of NCG used in this study was determined based on previous research (14), and the NCG used was obtained from Anhui Pusheng Pharmaceutical, a commercial company.

The sheep were housed in a cleaned and disinfected facility with natural ventilation and programmed lighting schedule. They were fed twice a day at 7 am and 6 pm, with free access to feed and water throughout the experimental period. The dietary ingredients and their chemical composition are presented in Table 1. The methods and procedures used in this study were approved by the Ethics Committee of Jinzhou Medical University.

**Table 1:** Composition and nutrient levels of the experimental basal diet, (%, as-fed basis).

Ingredients, %	
Corn	44.50
Corn germ meal	2.00
Distiller’s dried grains with solubles	3.00
Bean meal	11.00
Peanut vines	30.00
Wheat middlings	3.00
Puffing urea	0.50
Stone powder	1.00
Sodium sulphate	1.00
Natrium bicarbonate	1.00
Salt	0.50
Vitamin and trace mineral premix^a^	0.50
Molasses	2.00
Total	100.00
**Calculated composition, %**	
Digestible energy, MJ/kg	11.81
Dry matter	88.41
Crude protein	14.63
Crude fiber	10.97
Ash	5.49
Ether extract	2.76
Neutral detergent fiber	29.18
Acid detergent fiber	19.61
Acid detergent lignin	0.22
Calcium	1.16
Total phosphorus	0.24
Lysine	0.58
Methionine	0.22

a Provided per kg of complete diet: CuSO_4_·5H_2_O 18 g, FeSO_4_·H_2_O 82 g, ZnSO_4_·7H_2_O 90 g, MnSO_4_·H_2_O 85 g, CoSO_4_·7H_2_O 0.95 g, KI 6 g, Na_2_SeO_3_ 5 g, vitamin A 36,000 KIU/kg, vitamin D_3_ 7,500 KIU/kg, vitamin E 32,000 IU/kg, nicotinamide 38,000 mg/kg, vitamin H 42 mg/kg.

### Data collection and sampling

#### Growth performance analysis

The individual body weights of the sheep were recorded weekly to calculate the average daily gain (ADG). The feed intake of each pen was measured weekly to calculate the average daily feed intake (ADFI). Feed efficiency was calculated as the ratio of ADG to ADFI (G:F).

#### Hindgut SCFA analysis

On the final day of the experiment, four sheep were randomly selected from each group to obtain fresh stool samples for hindgut SCFA analysis using an Agilent Technologies Inc. 7,890-5,977 GC/MS (Agilent Technologies Inc., CA, United States). Data were manually processed after exportation from the instrument. The SCFA content of each sample was calculated as μg/mg = (sample concentration read by the instrument × final constant volume of the sample × the dilution factor)/sample weight.

#### Hindgut microbiota analysis

Fresh stool samples obtained above were immediately placed in iceboxes and transported to the laboratory. Total DNA was extracted from the microbial community using the E.Z.N.A.^®^ Soil DNA Kit (Omega Bio-Tek, Norcross, GA, United States) following the kit’s instructions. The quality of DNA extraction was assessed through 1% agarose gel electrophoresis, and the DNA density and purity were measured using the NanoDrop 2000 UV–vis spectrophotometer (Thermo Scientific, United States). The V3-V4 variable region of the *16S rRNA* gene was amplified by PCR using primers 338F (5′-ACTCCTACGGGAGCAGCAG-3′) and 806R (5′-GGACTACHVGGGTWTCTAAT-3′). Sequencing was performed using the Illumina MiSeq PE300/Novaseq PE250 platform. The original sequencing sequences were subjected to quality control using Fastp software (version 0.20.0). Line joining was performed using FLASH software (version 1.2.7) and UPARSE software (version 7.1). The operational taxonomic units (OTUs) were clustered at a 97% similarity level, and chimeric sequences were removed. The RDP Classifier (version 2.2) was used to annotate the species classification of each sequence, with comparisons made in the SILVA *16S rRNA* database (V138) using a comparison threshold of 70%. Alpha-diversity was estimated using the Chao1, Shannon, and Simpson indices, while beta-diversity was assessed using the *p*-value of Adonis analysis. The LEfSe analysis was performed using LEfSe software, with a linear discriminant analysis score threshold set at 3.5.

### Statistical analysis

Data on growth performance, hindgut SCFA contents, alpha-diversity of hindgut microbiota, and differences in hindgut microbiota at the genus level were analyzed using the student’s *t*-test procedure in SAS software (SAS Inst. Inc., Cary, NC, United States). The normality of the data was assessed using the Shapiro–Wilk test and QQ plots. Beta-diversity of the hindgut microbiota was analyzed using Adonis analysis. Spearman analysis was conducted to evaluate correlations among hindgut microbiota and hindgut SCFA contents. The results were presented as means ± standard deviation, with a probability value below 0.05 considered statistically significant.

## Results

Dietary supplementation of NCG had no significant effects on the growth performance of mutton sheep. The final body weight, ADG, ADFI, and feed efficiency did not differ among groups (Table 2).

**Table 2:** Growth performance of mutton sheep as affected by N-carbamylglutamate supplementation.

Items	CON^a^	TRT^b^
Initial BW, kg	30.11 ± 0.24	29.96 ± 0.25
Final BW, kg	40.76 ± 1.24	41.10 ± 1.61
ADG, g/day	177.50 ± 21.33	185.63 ± 26.96
ADFI, g	84.84 ± 8.28	85.93 ± 3.11
Feed efficiency	2.09 ± 0.25	2.16 ± 0.31

BW, body weight; ADG, average daily gain; ADFI, average daily feed intake. Results were presented as mean ± standard deviation.

a CON was defined as mutton sheep fed with basal diet.

b TRT was defined as mutton sheep fed with basal diet supplemented with 0.12% N-carbamylglutamate.

The contents of acetic acid (*p* < 0.05) and hexanoic acid (*p* < 0.05) in the hindgut of mutton sheep fed with an NCG-containing diet were lower than those fed with a basal diet. However, those fed with an NCG-containing diet presented a higher content of propanoic acid in the hindgut (*p* < 0.05) (Table 3).

**Table 3:** Contents of short-chain fatty acids from hindgut as affected by N-carbamylglutamate supplementation.

Items, μg/mg	CON^a^	TRT^b^
Acetic acid	39.60 ± 6.56^a^	29.31 ± 2.19^b^
Butyric acid	4.05 ± 0.36	3.87 ± 0.33
Propanoic acid	5.94 ± 1.10^b^	7.65 ± 0.59^a^
Isobutyric acid	0.47 ± 0.37	0.67 ± 0.13
Pentanoic acid	1.40 ± 0.45	1.47 ± 0.27
Isopentanoic acid	0.79 ± 0.52	0.85 ± 0.17
Hexanoic acid	0.56 ± 0.15^a^	0.19 ± 0.13^b^
Isohexanoic acid	0.70 ± 0.50	0.46 ± 0.22

Results were presented as mean ± standard deviation. ^a,b^Different superscripts within a row indicate a significant difference (*p* < 0.05).

a CON was defined as mutton sheep fed with basal diet.

b TRT was defined as mutton sheep fed with basal diet supplemented with 0.12% N-carbamylglutamate.

NCG supplementation did not affect the Shannon, Simpson, Ace, and Chao1 indexes of the hindgut microbiota (Table 4).

**Table 4:** Alpha-diversity of hindgut microbiota as affected by N-carbamylglutamate supplementation.

Items	CON^a^	TRT^b^
Shannon index	4.11 ± 0.67	4.04 ± 0.49
Simpson index	0.05 ± 0.04	0.05 ± 0.04
Ace index	478.61 ± 52.87	439.96 ± 52.12
Chao1 index	478.23 ± 58.37	439.52 ± 54.04

Results were presented as mean ± standard deviation.

a CON was defined as mutton sheep fed with basal diet.

b TRT was defined as mutton sheep fed with basal diet supplemented with 0.12% N-carbamylglutamate.

Spearman correlation analysis revealed that the abundance of *Lachnoclostridium* was positively correlated with the contents of acetic acid (*p* < 0.05) and hexanoic acid (*p* < 0.05), but negatively correlated with propanoic acid (*p* < 0.01). Additionally, the abundance of *Lachnospiraceae_NK3A20_group* and *Parasutterella* were positively correlated with the content of propanoic acid (*p* < 0.05), while being negatively correlated with acetic acid (*p* < 0.05) and hexanoic acid (*p* < 0.05) (Table 5).

**Table 5:** Spearman correlation analysis among hindgut microbiota and hindgut short-chain fatty acid contents.

Variables	Acetic acid	Propanoic acid	Hexanoic acid
*Unclassified_f_Lachnospiraceae*	0.578	−0.651	0.578
*Lachnociostridium*	0.735^*^	−0.880^**^	0.735^*^
*Lachnospiraceae_NK3A20_group*	−0.879^**^	0.733^*^	−0.879^**^
*Parasutterella*	−0.771^*^	0.843^**^	−0.771^*^

^*^p < 0.05, ^**^p < 0.01.

**Figure 1:**
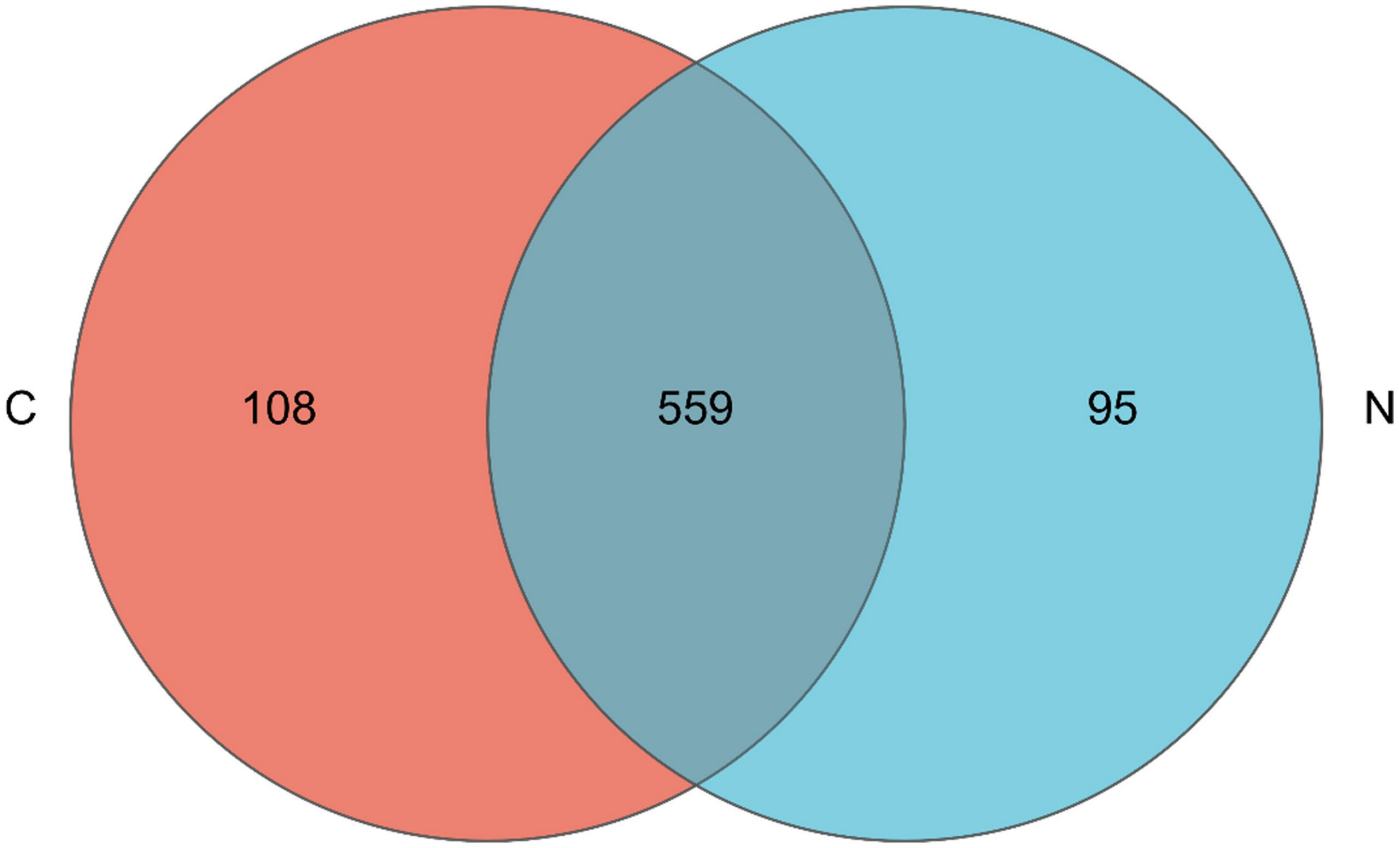
Venn diagram for hindgut microbiota of mutton sheep. Group C was defined as the sample from mutton sheep fed with basal diet. Group N was defined as the sample from mutton sheep fed with basal diet supplemented with 0.12% N-carbamylglutamate.

The Venn diagram showed that 559 bacterial OTUs were shared among groups, while 108 bacterial OTUs were unique in the hindgut microbiota of mutton sheep fed with a basal diet; 95 bacterial OTUs were unique in the hindgut microbiota of mutton sheep fed with an NCG-containing diet (Figure 1).

**Figure 2:**
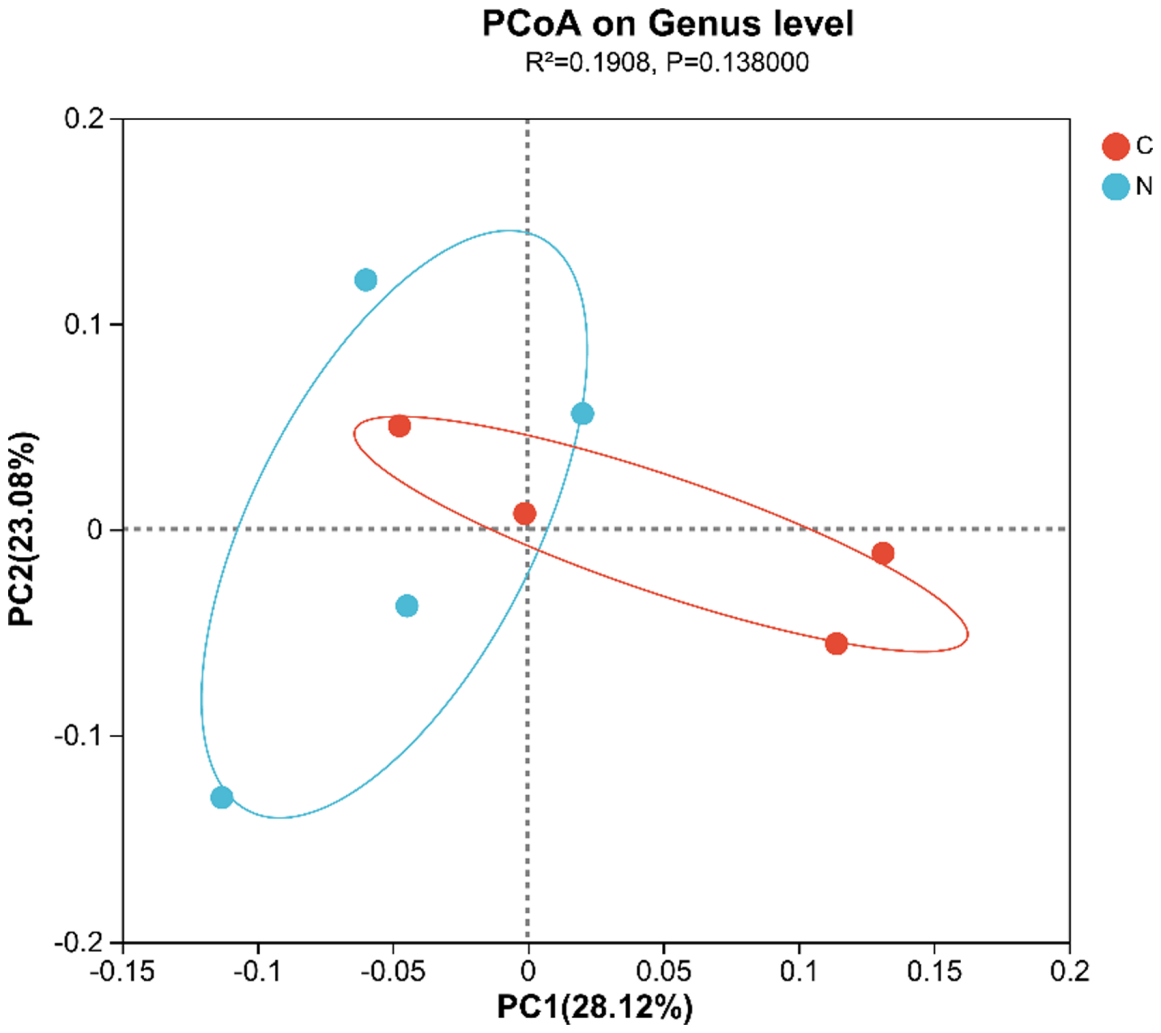
Differential analysis of the hindgut microbiota composition of mutton sheep, PCoA plot based on unweighted UniFrac distance. Group C was defined as the sample from mutton sheep fed with basal diet. Group N was defined as the sample from mutton sheep fed with basal diet supplemented with 0.12% N-carbamylglutamate.

Beta-diversity, presented in a PCoA plot based on unweighted UniFrac distance, displayed that the samples from all groups did not separate, indicating a similar hindgut microbiota community composition between groups (Figure 2).

**Figure 3:**
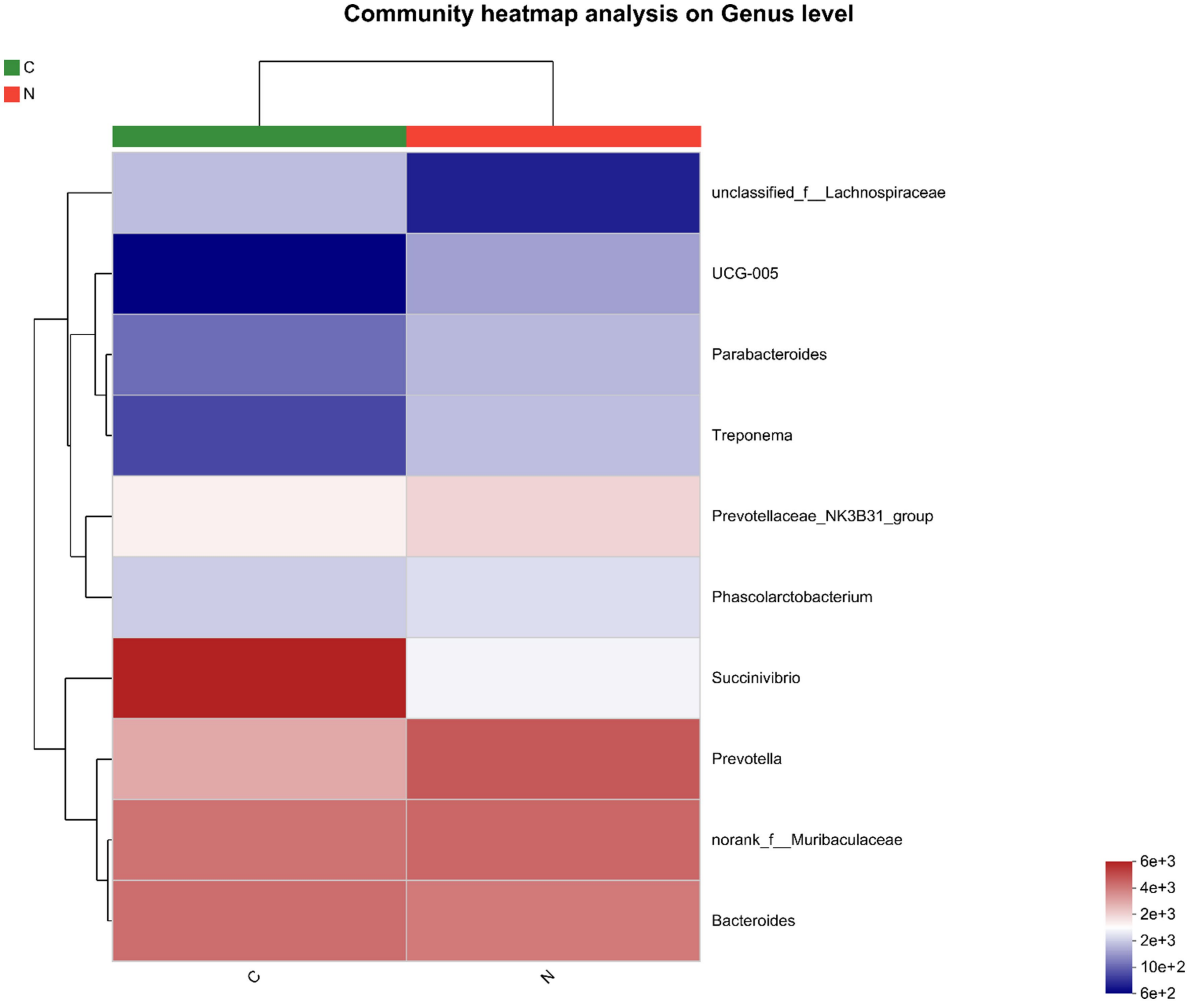
Heatmap of the ten most abundant bacteria of hindgut microbiota of mutton sheep on genus level. Group C was defined as the sample from mutton sheep fed with basal diet. Group N was defined as the sample from mutton sheep fed with basal diet supplemented with 0.12% N-carbamylglutamate.

The ten most predominant bacteria in the hindgut were *unclassified_f_Lachnospiraceae*, *UCG-005*, *Parabacteroides*, *Treponema*, *Prevotellaceae_NK3B31_group*, *Phascolarctobacterium*, *Succinivibrio*, *Prevotella*, *norank_f_Muribaculaceae*, and *Bacteroides* (Figure 3).

**Figure 4:**
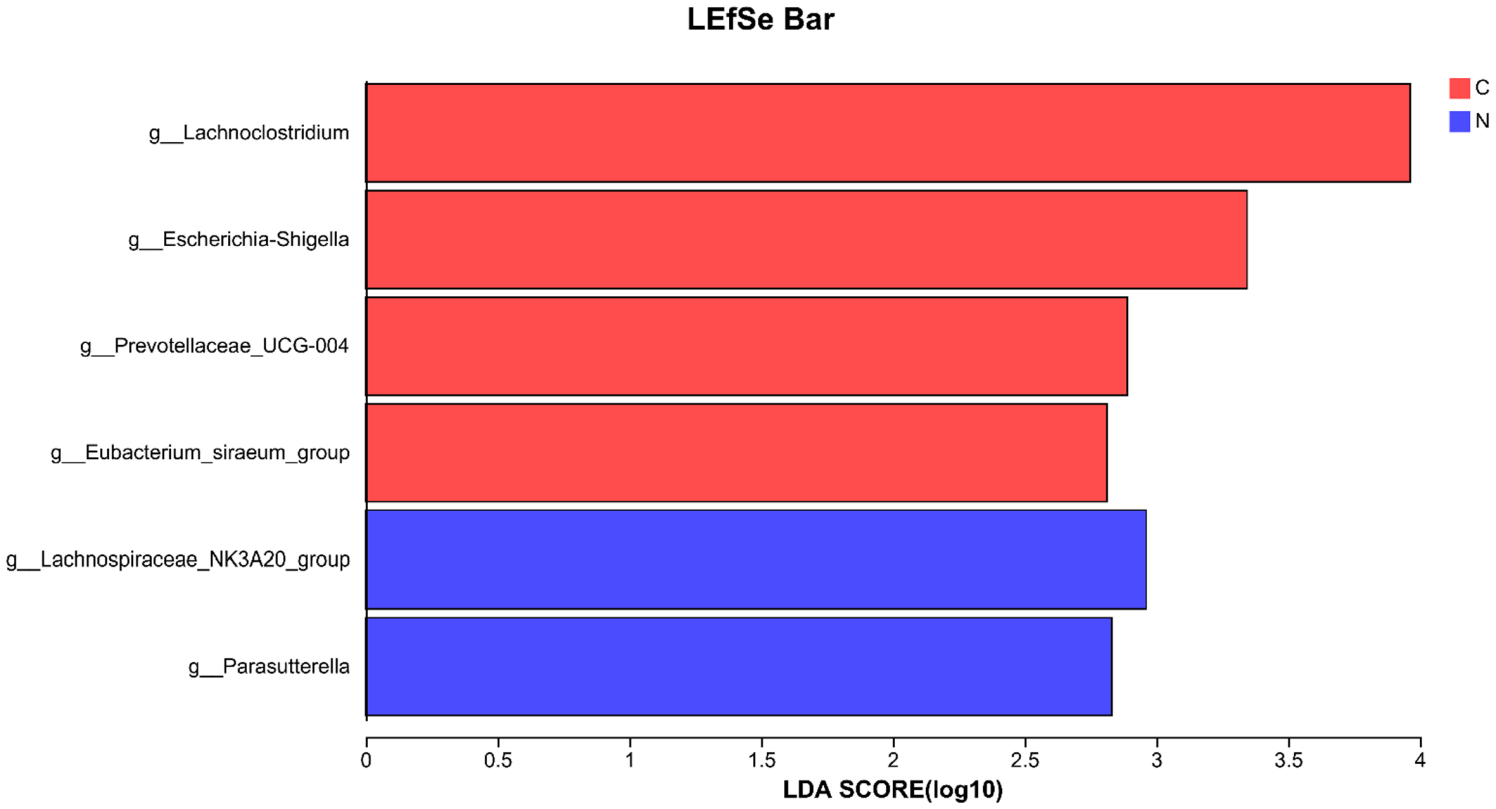
Biomarker of hindgut microbiota on genus level in different groups. The lineages in the values of least discriminant analysis higher than 3.5 are displayed. Group C was defined as the sample from mutton sheep fed with basal diet. Group N was defined as the sample from mutton sheep fed with basal diet supplemented with 0.12% N-carbamylglutamate.

Four genera (*Lachnoclostridium*, *Escherichia-Shigella*, *Prevotellaceae_UCG-004*, and *Eubacterium_siraeum_group*) with LDA scores <3.5 were identified as biomarkers for the hindgut of mutton sheep fed with a basal diet at the genus level. Additionally, two genera (*Lachnospiraceae_NK3A20_group* and *Parasutterella*) with LDA scores <3.5 were identified as biomarkers for the hindgut of mutton sheep fed with a basal diet supplemented with NCG at the genus level (Figure 4).

**Figure 5:**
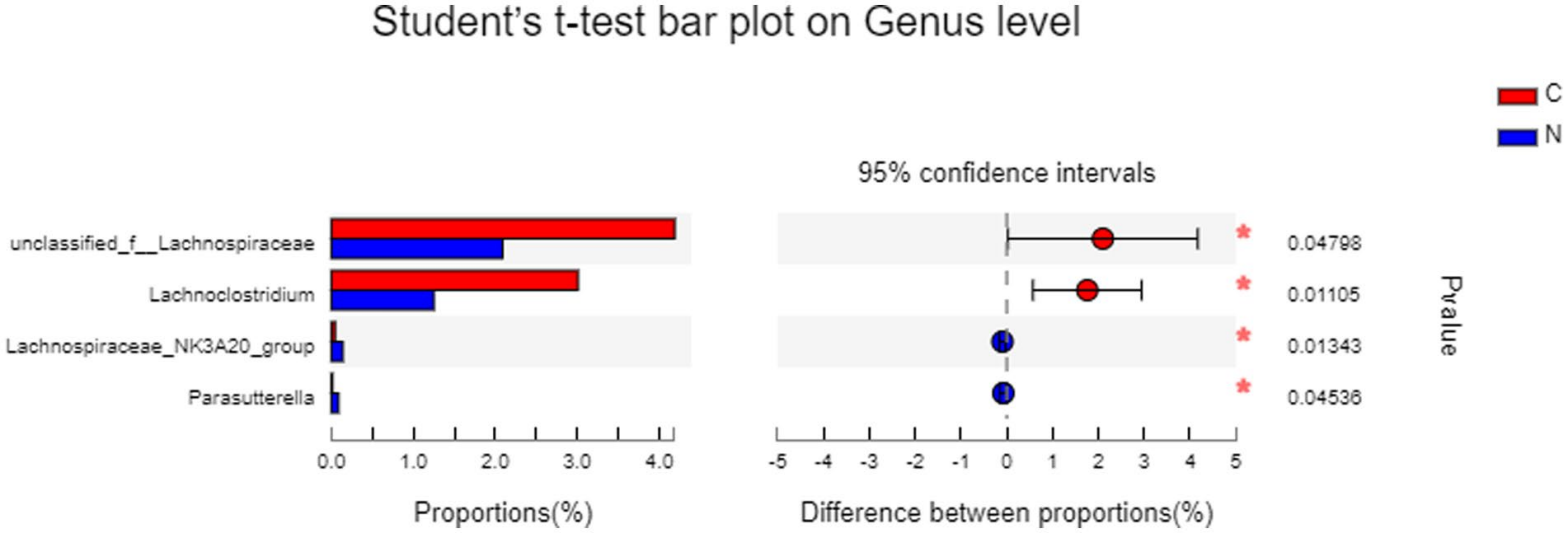
Statistical difference of hindgut microbiota between groups analyzed by *t*-test on genus level. Group C was defined as the sample from mutton sheep fed with basal diet. Group N was defined as the sample from mutton sheep fed with basal diet supplemented with 0.12% N-carbamylglutamate.

Statistical differences in hindgut microbiota between groups, analyzed by *t*-test on genus level, revealed that dietary supplementation of NCG significantly increased the abundance of *Lachnospiraceae_NK3A20_group* (*p* < 0.05) and *Parasutterella* (*p* < 0.05), while decreasing *unclassified_f_Lachnospiraceae* (*p* < 0.05) and *Lachnoclostridium* (*p* < 0.05) (Figure 5).

## Discussion

The hindgut microbiota encompasses a diverse array of organism communities that exert profound influences on the host’s development and metabolism. Preceding research has unveiled the modulatory potential of NCG upon the intestinal microbiota (15). Observations by Zhang et al. (16) demonstrated that dietary NCG supplementation ameliorated the diminished abundance of *Clostridium*, *Lactobacillus*, and *Streptococcus* within the colonic mucosa of lambs beset by intrauterine growth restriction. Likewise, Li et al. (17) noted that NCG supplementation invoked an elevation in *unidentified_Prevotellaceae* coupled with a decrease in *Acetitomaculum*, *Marvinbryantia*, and *unidentified_Christensenellaceae* within the fecal microbiome of heat-stressed lactating dairy cows. In line with this, our investigation revealed a consistent pattern of effects following NCG supplementation. Specifically, there was a noteworthy reduction in the abundance of *unclassified_f_Lachnospiraceae* and *Lachnoclostridium*, coupled with an increase in the abundance of *Lachnospiraceae_NK3A20_group* and *Parasutterella* within the fecal samples. Of particular significance, our analysis identified *Lachnospiraceae_NK3A20_group* and *Parasutterella* as distinctive biomarkers in mutton sheep that were fed the basal diet supplemented with NCG. The significance of these findings is underscored by the role of *Lachnospiraceae_NK3A20_group*, a recognized probiotic strain that actively participates in the production of SCFA (18). In parallel, the presence of *Parasutterella* stands as a reliable marker, indicating the presence of a health-promoting core microbiome in fecal compositions (19). Furthermore, the inverse correlation between the abundance of *unclassified_f_Lachnospiraceae* and the host’s immune status has been previously established (20). Notably, the heightened presence of *Lachnoclostridium* has been linked to instances of colorectal adenoma and cancer (21). Therefore, founded upon these robust findings, we proffer the notion that NCG supplementation exerts a favorable influence upon the composition of the hindgut microbiota. This influence is distinctly characterized by the augmentation of beneficial bacterial populations and the concomitant reduction of pathogenic strains.

SCFA serves as pivotal metabolites synthesized through the fermentation process carried out by the gut microbiota within the hindgut. These SCFAs substantially contribute to the overall health of the intestinal environment (22). In the context of our study, the administration of an NCG-enriched diet to mutton sheep resulted in elevated propanoic acid levels, concomitant with reduced acetic acid and hexanoic acid levels within the hindgut. Furthermore, the Spearman correlation analysis conducted within this study unveiled the intricate interplay between the abundance of specific bacterial genera and SCFAs content. These findings find support in the work of Rico et al. (23), who explored the interrelationship between the structure of the microbiome and fatty acid production stemming from cellulosic feedstocks in bison rumens. They discerned a negative correlation between the levels of acetic acid and the abundance of *Lachnospiraceae_NK3A20_group*. Similarly, in another study, Yi et al. (24) scrutinized the effects of the dietary concentrate-to-forage ratio on the bacterial community composition and metabolome within the rumens of yaks. They documented a positive correlation between propionate acid levels and the abundance of *Lachnospiraceae_NK3A20_group*. Therefore, we speculate that alterations in the SCFAs contents within the hindgut are intricately linked to shifts in the composition of the intestinal microbiota.

However, despite the discernible regulatory effects of dietary NCG supplementation on the composition of the hindgut microbiota and the contents of SCFA, no discernible discrepancies in growth performance were observed among the groups. This finding aligns with the outcomes of a prior study conducted by Sun et al. (25), wherein Holstein bulls were subjected to a daily regimen of NCG-infused diet, yet no notable impacts on body weight were discerned. The growth performance of animals represents an indicator influenced by a multitude of factors. As highlighted by Cai et al. (26), the augmentation of rumen fermentation in heat-stressed goats can be effectively achieved through the supplementation of exogenous probiotics, which serve to improve growth performance. Furthermore, Wang et al. (27) established that the introduction of organic acids can enhance the growth performance of lambs by enhancing their antioxidant ability. Consequently, our speculation rests on the premise that the enhancement of hindgut microbiota composition and SCFA contents, while influential, may not suffice to catalyze improvements in the growth performance of mutton sheep.

To conclude, our findings underscore the prospective utility of NCG as a dietary adjunct capable of modulating hindgut SCFA contents and the composition of the hindgut microbiota in mutton sheep. However, further investigations are warranted to delve into the underlying mechanisms at play and to comprehensively assess the enduring impacts of NCG supplementation on animal health and performance over the long term.

## Data availability statement

The datasets presented in this study can be found in online repositories. The names of the repository/repositories and accession number(s) can be found at: https://figshare.com/, https://doi.org/10.6084/m9.figshare.21608295.v1.

## Ethics statement

The animal study was approved by the methods and procedures used in the present study were approved by the Ethics Committee of Jinzhou Medical University. The study was conducted in accordance with the local legislation and institutional requirements.

## Author contributions

WM and MY: conceptualization and methodology. SC: data collection. WM: writing—original draft preparation. CW: investigation, supervision, and writing—reviewing and editing. All authors contributed to the article and approved the submitted version.
